# A topology-marginal composite likelihood via a generalized phylogenetic pruning algorithm

**DOI:** 10.1186/s13015-023-00235-1

**Published:** 2023-07-31

**Authors:** Seong-Hwan Jun, Hassan Nasif, Chris Jennings-Shaffer, David H Rich, Anna Kooperberg, Mathieu Fourment, Cheng Zhang, Marc A Suchard, Frederick A Matsen

**Affiliations:** 1 Department of Biostatistics and Computational Biology, University of Rochester, Rochester, USA; 2 Department of Statistics, University of Washington, Seattle, USA; 3 Public Health Sciences Division, Fred Hutchinson Cancer Research Center, Seattle, WA USA; 4 Australian Institute for Microbiology and Infection, University of Technology Sydney, Ultimo, NSW Australia; 5 School of Mathematical Sciences and Center for Statistical Science, Peking University, Beijing, China; 6 Department of Human Genetics, University of California, Los Angeles, USA; 7 Department of Computational Medicine, University of California, Los Angeles, USA; 8 Department of Biostatistics, University of California, Los Angeles, USA; 9 Department of Genome Sciences, University of Washington, Seattle, USA; 10 Howard Hughes Medical Institute, Fred Hutchinson Cancer Research Center, Seattle, Washington USA; 11 Computational Biology Program, Fred Hutchinson Cancer Research Center, 1100 Fairview Ave. N., Mail stop: S2-140, Seattle, WA 98109-1024 USA

## Abstract

Bayesian phylogenetics is a computationally challenging inferential problem. Classical methods are based on random-walk Markov chain Monte Carlo (MCMC), where random proposals are made on the tree parameter and the continuous parameters simultaneously. Variational phylogenetics is a promising alternative to MCMC, in which one fits an approximating distribution to the unnormalized phylogenetic posterior. Previous work fit this variational approximation using stochastic gradient descent, which is the canonical way of fitting general variational approximations. However, phylogenetic trees are special structures, giving opportunities for efficient computation. In this paper we describe a new algorithm that directly generalizes the Felsenstein pruning algorithm (a.k.a. sum-product algorithm) to compute a composite-like likelihood by marginalizing out ancestral states and subtrees simultaneously. We show the utility of this algorithm by rapidly making point estimates for branch lengths of a multi-tree phylogenetic model. These estimates accord with a long MCMC run and with estimates obtained using a variational method, but are much faster to obtain. Thus, although generalized pruning does not lead to a variational algorithm as such, we believe that it will form a useful starting point for variational inference.

**Supplementary Information:**

The online version contains supplementary material available at 10.1186/s13015-023-00235-1.

## Introduction

Statistical phylogenetics is largely divided into maximum-likelihood based and Bayesian posterior-based approaches. The former searches for a tree that yields highest likelihood for the observed sequencing data, while the latter is typically approached using Markov chain Monte Carlo (MCMC) sampling to estimate the posterior probabilities of trees given the observed sequencing data. Both approaches involve exploration of the tree space using local tree rearrangements, such as nearest neighbor interchange or subtree pruning and regrafting [1], followed by computation of the likelihood of the sequence data given the tree using Felsenstein’s pruning algorithm [2]. The Felsenstein pruning algorithm prunes out the ancestral states at the internal nodes of the tree and is the engine driving the advances in statistical phylogenetics, allowing estimation of branch lengths of a tree as well as the parameters of the evolutionary models. Specifically, the two-pass version of the algorithm allows for constant-time updates to branch lengths and local tree structures. In this paper, we propose a generalization of the two-pass Felsenstein pruning algorithm that marginalizes uncertain tree structures as well as the ancestral states, with a long-term goal of bringing efficient optimization strategies from maximum-likelihood phylogenetics to Bayesian inference.

To begin to appreciate the challenges of Bayesian phylogenetics, we start with an overview of likelihood-based phylogenetic models (see the Background and Notation section for a full development). Assume that we are given a multiple sequence alignment [3, 4] of DNA sequences as data ${\textbf{Y}}$ that maps a sequence of molecular characters (i.e. DNA bases) to each leaf of the phylogenetic tree that generated it. This alignment is organized in terms of a list of *sites* such that the differences between sites are assumed to arise only due to substitution of one DNA base for another along the course of evolution. Specifically, per-site sequence change is formulated in terms of continuous-time Markov chain (CTMC) models of DNA sequence evolution along the branches of the tree, where the time parameter in the CTMC is called *branch length*. The CTMC may also have other parameters, such as the rate of change from one DNA base to another. For this paper, a *(phylogenetic) tree* is defined to be a rooted bifurcating tree structure $\tau$ with leaf labels that has been equipped with branch lengths on every edge. We follow common practice by using the word *topology* to describe the discrete component of this model, namely the tree without branch lengths.

We make the typical independence-across-sites assumption for evolutionary processes conditioned on the tree, which enables efficient likelihood computation via a dynamic programming approach that integrates out the unobserved molecular characters (*ancestral states*) at all of the internal nodes. This approach is called the *pruning algorithm* [2] in phylogenetics, which is reviewed below, and is also known as the sum-product algorithm or belief propagation in other settings [5]. It enables linear-complexity calculation in the number of sequences for the phylogenetic likelihood $p({\textbf{Y}}\mid \tau , {\varvec{\theta }})$, where $\tau$ is the topology and ${\varvec{\theta }}$ is a corresponding vector of branch lengths, as well as constant-complexity updates for local modifications. Assume we are given a prior $p(\tau , {\varvec{\theta }})$ on phylogenetic trees. We assume here that the prior factors into two easily-calculated terms: the prior $p(\tau )$ on topologies and the prior $p({\varvec{\theta }}\mid \tau )$ on branch lengths given a topology.

Recent work has fit reduced-dimension probabilistic models to the topological posterior [6–9]. Briefly, these methods break topologies into building blocks such that probabilistic models on these building blocks can be translated into probabilistic models on whole topologies themselves. We have shown that this translation provides a flexible distribution on topologies with good inductive biases [8].

**Fig. 1:**
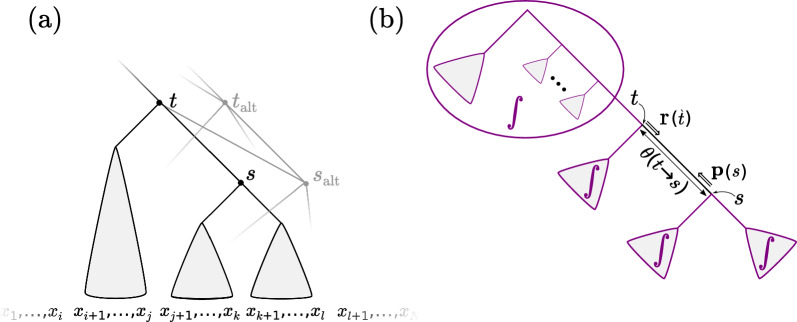
A preview of the core components of the algorithm to give intuition; concepts will be introduced in the text. **a** The subsplit DAG, which encodes a collection of phylogenetic tree topologies on leaves $x_1, \ldots , x_N$. One such topology is partially shown in black, with alternate topologies indicated with gray lines. The nodes of this DAG are uniquely associated with “subsplits” that give the bipartition of taxa below them (subsplits corresponding to alternate topologies are marked with $\cdot _{\text {alt}}$). For example, the subsplit *t* is $(\{x_{i+1}, \ldots , x_j\},\{x_{j+1}, \ldots , x_l\})$. Edges go between compatible subsplits, such as between *t* and *s*, and are directed towards the leaves. **b** Overview of method: given an edge of the DAG we integrate ($\int$) out all of the topologies in the DAG that contain the DAG edge $t \rightarrow s$. Branch lengths $\theta$ are associated to DAG edges. We perform efficient inference using “rootward” $\textbf{r}$ and “leafward” ${\textbf{p}}$ partial likelihood vectors marginalized over unknown structure of trees encoded in the DAG

One can think of these reduced-dimension models in terms of a structure we call a *subsplit directed acyclic graph* or *subsplit DAG* (introduced below). One can think of the nodes of the subsplit DAG as comprising the union of substructures, called *subsplits*, of a collection of topologies. The edges between these nodes represent compatibility of substructures (Fig. 1a). We arrive at a probability distribution on topologies by attaching probabilities to the edges of the DAG.

Stated in these terms, in [9] we used a variational approach, with modern yet general-purpose gradient estimators, to fit continuous parameters (i.e. edge probabilities and branch length distributions) to the subsplit DAG. This variational approach results in an excellent approximation to the phylogenetic posterior distribution, and converges in relatively few iterations. However, this variational approach struggles to be time-competitive with classical random-walk MCMC because of the stochasticity of the gradient estimator as well as the cost of evaluating the gradient of the phylogenetic likelihood function.

In this work, we begin addressing these difficulties via a new algorithm that performs dynamic programming directly on the subsplit DAG via a generalization of the Felsenstein algorithm. This *generalized pruning* (GP) algorithm marginalizes a likelihood function over ancestral states and topologies at the same time (Fig. 1). By forming a new type of partial likelihood vector that integrates out all of the topologies in the support that contain a given DAG edge, we are able to perform constant-time updates to the branch length associated with that structure.

This generalized pruning algorithm based on integrated partial likelihood vectors relies on several modeling approximations. First, the likelihood we use is a composite-like model likelihood marginalized over topologies $\tau$:

1 $$\begin{aligned} \prod _{k=1}^K \, \sum _{\tau \in {\mathcal {D}}} p_{{\varvec{\theta }}}(Y_k \mid \tau ) \, p(\tau ) \end{aligned}$$

where $Y_k$ is the *k*-th column of the sequence alignment ${\textbf{Y}}$ and ${\mathcal {D}}$ is a structure that contains many topologies described below. In contrast, exact likelihood computation marginalizes over the topologies, with each term being a product across sites. Second, we parameterize the continuous aspects of phylogenetic trees in a very simple way: one fixed branch length per DAG edge. This is unusual for Bayesian phylogenetics, in which one typically considers a distribution of branch lengths, however this style of modeling approximation achieves surprisingly good performance [10–12]. Furthermore, this is a necessary assumption for the efficient implementation of our GP algorithm, as anything else would require an integration over branch lengths. We use ${\varvec{\theta }}$ to denote the vector of these parameters.

This paper is focused on providing a complete description of the generalized pruning algorithm, as well as describing fast parameter estimation procedures for the composite-like model likelihood shown in Eq. (1). We perform experiments to demonstrate the efficacy of the procedures introduced in this paper, specifically comparing branch length estimation to a more established procedure [9], benchmarked in terms of computational effort. We emphasize that generalized pruning does not lead to a variational algorithm as such, however, we believe that it will form a useful companion for variational inference by providing initial parameter estimates.

We assume for this paper that the subsplit DAG is provided to the algorithm. Constructing this DAG is an interesting challenge in itself and the subject of ongoing research; we hope to use generalized pruning to infer the structure of the subsplit DAG using a procedure analogous to maximum-likelihood phylogenetic inference. However, one existing option is to build the subsplit DAG out of trees obtained by bootstrapping as practiced in maximum likelihood phylogenetics [9], or to use trees from an initial MCMC run [13].

## Background and notation

We begin by introducing common phylogenetics notation (mostly following [14]) and the notion of subsplits as buildings blocks for modeling tree structures.

### Setup for likelihood-based phylogenetics

We use the word *taxon* (plural *taxa*) to describe an entity associated with a molecular sequence. In classical evolutionary phylogenetics, taxa are commonly species, although they could be other entities such as samples of viruses. We assume that a taxon set *X* is given, and that we have a lexicographic order on it. Also assume that we are given a sequence alignment on our taxon set *X*: an arrangement of the molecular sequences for *X* into a rectangular array such that sequence differences between sites in a single column are assumed to be due to point mutation [4, 15]. Here we focus on rooted bifurcating trees, and as mentioned above use the terminology *topology* to refer to a rooted bifurcating tree structure with the tips of the tree being labeled in a 1-to-1 fashion with the set *X*.

We use $N = |X|$ to denote the cardinality of the taxon set under consideration. The observed sequences are denoted by ${\textbf{Y}}$. We let $\Sigma$ denote the set of states in the sequences. If the sequences are DNA, $\Sigma$ is the set of nucleotide bases $\{\texttt{A}, \texttt{C}, \texttt{G}, \texttt{T}\}$; our implementation is specialized to this case. The length of the DNA sequences is denoted by *M*. We denote the *m*-th site over all taxa (i.e. the *m*-th column of the alignment) by ${\textbf{Y}}^{m} \in \Sigma ^N$. Topologies are denoted by $\tau$. The collection of all branch length parameters is denoted by ${\varvec{\theta }}$ with individual branch length by $\theta$. The likelihood of the observed sequences is denoted by ${\mathbb {P}}({\textbf{Y}}\mid \tau , {\varvec{\theta }})$, which under the standard site independence assumption can be expressed as

2 $$\begin{aligned} {\mathbb {P}}({\textbf{Y}}\mid \tau , {\varvec{\theta }}) = \prod _{m=1}^{M} {\mathbb {P}}({\textbf{Y}}^{m} \mid \tau , {\varvec{\theta }}). \end{aligned}$$

The phylogenetic model underlying the likelihood computation is typically a continuous time Markov chain (CTMC) evolving along the branches. The rate matrix of a CTMC is denoted by $\textbf{Q}$, which yields the transition matrix via matrix exponentiation, denoted ${\textbf{P}}$:

$$\begin{aligned} {\textbf{P}}({\theta }) = \exp (\theta \textbf{Q}). \end{aligned}$$

The transition matrix plays a key role in the likelihood calculation. We follow the English-typical convention that probability transition matrices are right-stochastic, so that the (*i*, *j*)-th entry of ${\textbf{P}}$ is ${\mathbb {P}}(j \mid i)$ for $i, j \in \Sigma$. We denote transposition of vectors and matrices by $^\top$ (in contrast to [14], which uses $'$). For brevity of notation, we may omit $\theta$ when referencing a specific entry of the transition matrix.

For simplicity of exposition and implementation, we assume the Jukes-Cantor model for DNA sequences, under which there are no CTMC model parameters other than branch lengths. Additional CTMC model parameters could be added and fit in a maximum-likelihood sense without much difficulty, but given that such parameter fitting is now standard, we focus on our novel tree-marginalization procedure. However, in the mathematical exposition we do not assume that the probability transition matrices are symmetric as they are in the Jukes-Cantor model.

### Likelihood calculation over a tree using a two-pass algorithm

We briefly describe the two-pass version of the Felsenstein pruning algorithm [2, 14, 16–18] over a single tree using the notion of *partial likelihood vectors* (PLVs). For the rest of this section, **we will compute the likelihood of a single site** *m* without further specification, such that what we called ${\textbf{Y}}^m$ will now be called ${\textbf{Y}}$. We will return to the multiple-site case in the section “Composite-like marginal likelihood.”

We follow the exposition and notation in [14], except that we express partial likelihood vectors in terms of subtrees, because in our setting we will deal with many trees and cannot unambiguously describe the algorithm in terms of nodes of a given fixed tree. We also compute partial likelihood vectors at a slightly different location on the tree. For an internal node *v*, $Y_v \in \Sigma$ will denote the state of *v*. We use the word “leafward” to refer to the direction in the tree towards the leaves; if the tree is displayed with the root on top and the leaves hanging down, leafward is down. ${\textbf{Y}}_{\lfloor v\rfloor }$ will denote the sequences leafward of *v*. The direction towards the root of the tree will be called “rootward” and we let ${\textbf{Y}}_{\lceil v\rceil } = {\textbf{Y}}{\setminus } {\textbf{Y}}_{\lfloor v\rfloor }$.

Assume we have a topology $\tau$ on all of the sequences ${\textbf{Y}}$. Define $\tau ^{\downarrow }_v$ to be the topology with all the nodes leafward of *v*, including *v*. Define $\tau ^{\uparrow }_v$ to be the topology with all the nodes of the tree in the rootward direction of *v*, excluding *v*; that is, $\tau ^{\uparrow }_v$ is the topology on all of the nodes not in $\tau ^{\downarrow }_v$. The *i*-th element of the *p*- and *r*-PLVs at node *v* of the topology $\tau$ store

3 $$\begin{aligned} {{\textbf{p}}(\tau ^{\downarrow }_v)}_i&:= {\mathbb {P}}({\textbf{Y}}_{\lfloor v\rfloor } \mid \tau ^{\downarrow }_v, \, Y_v = i) , \end{aligned}$$

4 $$\begin{aligned} {\textbf{r}(\tau ^{\uparrow }_v)}_i&:= {\mathbb {P}}({\textbf{Y}}_{\lceil v\rceil },Y_{{\textsf{pa}}(v)} = i \mid \tau ^{\uparrow }_v) , \end{aligned}$$

where $i \in \Sigma$ and ${\textsf{pa}}(v)$ is the parent node of *v* (towards the root). Note that we describe ${\textbf{p}}$ and $\textbf{r}$ in terms of topologies on subsets of the taxon set, rather than simply a node *v*, which will become important below when we allow $\tau$ to vary. Equation (3) can be interpreted as the marginal likelihood of the observed sequences “below” *v*, conditioned on $Y_v = i$ for $i \in \Sigma$. Similarly, Eq. (4) stores the likelihood of the data “above” *v*, however, this time we have the joint likelihood of the observed sequences along with the state at the parent of *v*.

Note that for any *v*, the likelihood is given by the product of the *p*- and *r*-vectors:

$$\begin{aligned}&{\textbf{r}(\tau ^{\uparrow }_v)}^\top {\textbf{P}}(\theta _{{\textsf{pa}}(v),v}) {\textbf{p}}(\tau ^{\downarrow }_v) = {\mathbb {P}}({\textbf{Y}}\mid {\varvec{\theta }}, \tau ) \\&\quad = \sum _{i,j \in \Sigma } {\mathbb {P}}({\textbf{Y}}_{\lceil v\rceil }, Y_{{\textsf{pa}}(v)} = i \mid \tau ^{\uparrow }_v) \\&\qquad\qquad {\mathbb {P}}(Y_{{\textsf{pa}}(v)} = i, Y_v = j \mid {\varvec{\theta }}) \\&\qquad\qquad {\mathbb {P}}({\textbf{Y}}_{\lfloor v\rfloor } \mid \tau ^{\downarrow }_v, Y_v = j). \end{aligned}$$

Such a decomposition can be performed with respect to any internal node.

**Fig. 2:**
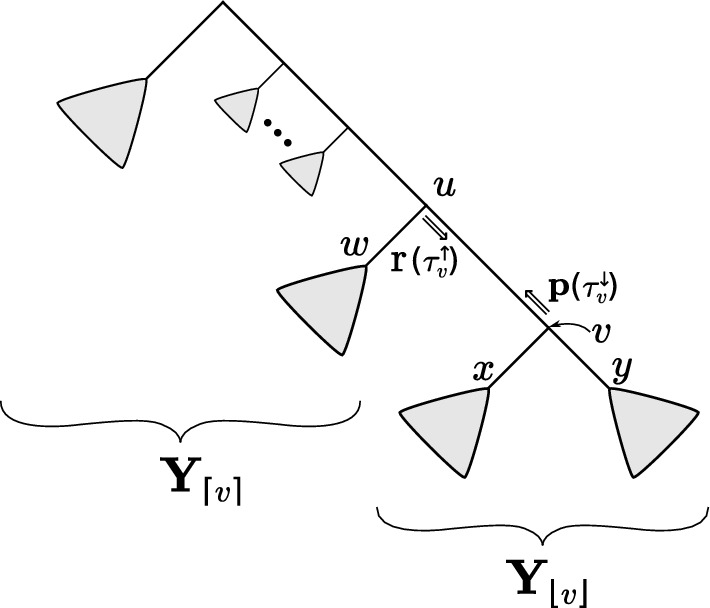
Notation for the two-pass likelihood calculation on a tree. Nodes are denoted with *u*, *v*, *w*, *x*, and *y*, and partial likelihood vectors are denoted with ${\textbf{p}}$ and $\textbf{r}$. The directions of double arrows indicate the flow of information to calculate the likelihood of a tree at a given edge

We now review the classical two-pass dynamic program showing how to calculate these partial likelihood vectors, mostly following [14] but with a slightly different formulation, which is more appropriate for our setting. Specifically, our $\textbf{r}$ vectors are our “upper partial” vectors, taking the role of their $\textbf{q}$ vectors, but calculated on the root-side rather than the leaf-side of a given edge.

Let *x* and *y* be the child nodes of *v* and $\theta _{v,x}, \theta _{v,y}$ denote the branch length between *v*, *x* and *v*, *y* respectively (Fig. 2). Dropping conditioning on tree structures and branch lengths for simplicity yields

$$\begin{aligned} {\mathbb {P}}({\textbf{Y}}_{\lfloor v\rfloor } \mid Y_v)&= \sum _{Y_x \in \Sigma } \sum _{Y_y \in \Sigma } {\mathbb {P}}({\textbf{Y}}_{\lfloor v\rfloor }, Y_x, Y_y \mid Y_v) \\&= \Bigg [ \sum _{Y_x \in \Sigma } {\mathbb {P}}(Y_x \mid Y_v) \, {\mathbb {P}}({\textbf{Y}}_{\lfloor x\rfloor } \mid Y_x) \Bigg ] \Bigg [\sum _{Y_y \in \Sigma } {\mathbb {P}}(Y_y \mid Y_v) \, {\mathbb {P}}({\textbf{Y}}_{\lfloor y\rfloor } \mid Y_y) \Bigg ]. \end{aligned}$$

In matrix notation,

5 $$\begin{aligned} {\textbf{p}}(\tau ^{\downarrow }_v) = \left( {\textbf{P}}(\theta _{v,x}) \, {\textbf{p}}(\tau ^{\downarrow }_x)\right) \circ \left( {\textbf{P}}(\theta _{v,y}) \, {\textbf{p}}(\tau ^{\downarrow }_y)\right) \end{aligned}$$

where $\circ$ denotes element-wise multiplication.

Now let *u* be the parent of *v* (Fig. 2). We have

$$\begin{aligned} {\mathbb {P}}({\textbf{Y}}_{\lceil y\rceil }, Y_v)&= {\mathbb {P}}({\textbf{Y}}_{\lfloor x\rfloor } | Y_v) \, {\mathbb {P}}({\textbf{Y}}_{\lceil v\rceil }, Y_v) \\&= \Bigg [\sum _{Y_x \in \Sigma } {\mathbb {P}}(Y_{x} | Y_v) {\mathbb {P}}({\textbf{Y}}_{\lfloor x\rfloor } | Y_x) \Bigg ] \Bigg [\sum _{Y_u \in \Sigma } {\mathbb {P}}(Y_v | Y_u) {\mathbb {P}}({\textbf{Y}}_{\lceil v\rceil }, Y_u) \Bigg ]. \end{aligned}$$

This recursion can be expressed in matrix form as

6 $$\begin{aligned} \textbf{r}(\tau ^{\uparrow }_y) = \left( {\textbf{P}}(\theta _{v,x}) {\textbf{p}}(\tau ^{\downarrow }_x) \right) \circ \left( {\textbf{P}}(\theta _{u,v})^\top \textbf{r}(\tau ^{\uparrow }_v) \right) . \end{aligned}$$

**Fig. 3:**
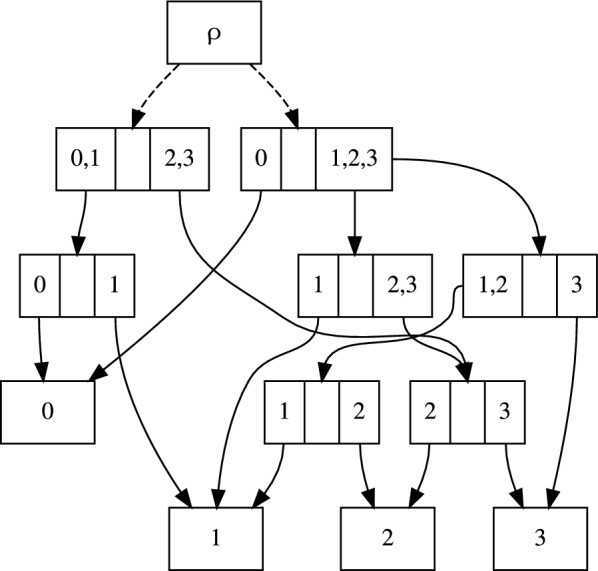
The subsplit DAG containing three trees on four taxa. Written in Newick [19] parenthetical notation these are ((0, 1), (2, 3)), (0, ((1, 2), 3)), and (0, (1, (2, 3))). We obtain a tree by choosing a single edge out of every “clade” (e.g. $\{1,2,3\}$) from each subplit (e.g. $\{\{0\},\{1,2,3\}\}$), and one of the dashed edges from the DAG root $\rho$

### The subsplit directed acyclic graph

In addition to the phylogenetic likelihood computation, the other main ingredient for our algorithm is a structure on which one can marginalize over tree structures. We call this structure the *subsplit directed acyclic graph* (*subsplit DAG*), which encodes a collection of tree topologies, $\mathcal {T}$ (Fig. 3). This structure can be equipped with edge probabilities to give a probability distribution on tree topologies, and with branch lengths or distributions thereof to give a probability distribution on phylogenetic trees. We use the subsplit DAG to develop a formulation of variational distributions on tree structures as previously expressed in different language [8, 9]. This new language is necessary for the more complex traversals required here.

To define the subsplit DAG, we need a few concepts as follows. A *clade* *W* is subset of the taxa *X*, which in the context of a topology $\tau$ identifies a subset of the taxa deriving from a common ancestor (i.e., monophyletic group). The order on taxa induces a total order on the clades. A *subsplit* $s = \{V, Z\}$ partitioning a clade *W* is an unordered pair of disjoint subclades of *W* such that $V \cup Z = W$. *V* and *Z* are called the clades of the subsplit *s*. We define subsplits in terms of unordered pairs for when we need to express a subsplit in terms of set operations, but always draw subsplits in lexicographic order in figures and call the lexicographically smaller clade the “left” clade and the lexicographically larger clade the “right” clade. We require that the two clades forming a subsplit are each non-empty except in two special cases: “leaf subsplits” $\{\{x\}, \emptyset \}$ for some $x \in X$, and the “universal ancestor (UA) subsplit” $\rho := \{X, \emptyset \}$. Given a subsplit $s = \{V, Z\}$, define *U*(*s*) to be $V \cup Z$, the set of taxa in the subsplit.

With the above definitions, we can define the subsplit DAG for a collection of topologies $\mathcal {T}$ as a graph with nodes being the set of subsplits for those topologies, and where there is an edge from *t* to *s* if *U*(*s*) corresponds to one of the two clades of *t*. (One can define a subsplit DAG in a more abstract way without referring to a set of topologies $\mathcal {T}$, but this definition is sufficient here.) For the purposes of this paper we assume that $\mathcal {T}$ is supplied. In the long run, and as described in the Discussion, our goal with generalized pruning is to enable algorithms that will allow us to infer the structure of the subsplit DAG.

We will also need a notion of a subsplit where we are focusing attention on one of the clades of the subsplit, which we will call a *subsplit-clade*. This is useful, for example, in describing a collection of edges descending from a single “side” of a subsplit (e.g. the two edges coming from clade $\{1,2,3\}$ in the subsplit $\{\{0\},\{1,2,3\}\}$ in Fig. 3). Given a subsplit *s* and a clade *Z* of *s*, we let $(s,{\underline{Z}})$ denote the subsplit-clade focusing attention on the clade *Z*. We use $\acute{s}$ to denote the left (i.e. lexicographically smaller) subsplit-clade of subsplit *s* and $\grave{s}$ to denote the right (i.e. lexicographically larger) subsplit-clade of subsplit *s*. We say a topology $\tau$ contains a subsplit $s=\{V, Z\}$ if *V*, *Z*, and $V \cup Z$ are all clades of the topology $\tau$. We denote the DAG on the subsplits by ${\mathcal {D}}$ and define $\rho$ to be the DAG root; we will also use $\rho$ to signify the subsplit at the DAG root: $\{X, \emptyset \}$. We say an edge $t \rightarrow s \in {\mathcal {D}}$ iff there is an edge $(t,{\underline{Z}}) \rightarrow s$ in the DAG for one of the clades *Z* of *t*. Similarly, we say an edge $t \rightarrow s \in \tau$ iff *s*, *t* are contained in $\tau$ and *s* appears as one of the clades *Z* of *t*. We can also say $\tau \in {\mathcal {D}}$ iff $t \rightarrow s \in {\mathcal {D}}$ for all $t \rightarrow s \in \tau$.

### Parameterizing the subsplit DAG

We can equip the subsplit DAG with parameters that turn it into a true probability distribution on phylogenetic trees [8, 9]. First, we have probability distributions for resolving a subsplit-clade $(t,{\underline{Z}})$, which we will write as ${\mathbb {P}}(s \mid (t,{\underline{Z}}))$, such that

$$\begin{aligned} {\mathbb {P}}(s \mid (t,{\underline{Z}}))&\ge 0, \\ \sum _{s: (t,{\underline{Z}}) \rightarrow s} {\mathbb {P}}(s \mid (t,{\underline{Z}}))&= 1. \end{aligned}$$

We can simplify notation by defining ${\mathbb {P}}(s \mid t)$ for an edge $t \rightarrow s$ of the DAG to be whichever version makes sense: for example if $\acute{t} \rightarrow s$, then ${\mathbb {P}}(s \mid t):= {\mathbb {P}}(s \mid \acute{t})$. Note that this is an abuse of notation because ${\mathbb {P}}(s \mid t)$ is not a normalized probability distribution across all possible *s* because they are allowed to resolve either clade of a given subsplit.

These conditional probabilities combine to give a normalized probability distribution on topologies [8]:

7 $$\begin{aligned} {\mathbb {P}}(\tau ) = \prod _{t\rightarrow s \in \tau } {\mathbb {P}}(s \mid t). \end{aligned}$$

Given probabilities on ${\mathbb {P}}(s \mid t)$, we can recursively compute the probability of a sub-topology descending from a subsplit. For example, imagine that we have two topologies $(\tau _1, \tau _2)$ on disjoint taxon subsets of *X*, and that *t* is the subsplit consisting of the taxa of $\tau _1$ for one clade and the taxa of $\tau _2$ for the other. Assume that the taxon set of $\tau _1$ is lexicographically smaller than that of $\tau _2$. There is a 1-to-1 correspondence between all such $(\tau _1, \tau _2)$ ordered pairs on those taxon sets and topologies $\tau _t$ on the union of the taxon sets: if we are given $(\tau _1, \tau _2)$ we simply join them together to make $\tau _t$. We express the conditional probability of such a topology recursively as

8 $$\begin{aligned} {\mathbb {P}}(\tau _t \mid t) = {\mathbb {P}}(s_1 \mid \acute{t}) \, {\mathbb {P}}(\tau _1 \mid s_1) \, {\mathbb {P}}(s_2 \mid \grave{t}) \, {\mathbb {P}}(\tau _2 \mid s_2), \end{aligned}$$

where each $s_i$ is the subsplit on the taxa appearing in subtrees $\tau _i$ for $i = 1, 2$. We utilize such recursive definitions of the tree probability in the development of generalized pruning algorithm.

However, in contrast to previous work [8, 9] in which these probabilities express an approximation to the posterior distribution, in this case they express a prior on tree topologies, which we will combine with a likelihood to get a posterior kernel. For this paper we assume that the topological prior can be expressed in terms of such ${\mathbb {P}}(s \mid t)$. This exact criterion is not strictly necessary, but we do need a way of computing it in terms of rootward and leafward components. Further details on prior computation are given in the section “Prior on subsplit parameters.”

As described above, we will attach a single branch length parameter to each edge of the DAG. The branch length for edges originating in the root node $\rho$ have no meaning and can be ignored.

The immediate goal of our work is to optimize these branch lengths via marginalization of tree topologies; the exact meaning of this will be made explicit in the next section.

## Methods

In this section we describe the two-pass generalized pruning (GP) algorithm. We assume a DAG ${\mathcal {D}}$ is given, and all statements about DAG edges are with respect to that given DAG. The two-pass GP algorithm can efficiently compute the marginal likelihood for a single site, where marginalization is over the topologies and the states of the internal nodes of the trees in ${\mathcal {D}}$. Utilizing the two-pass algorithm, we formulate the *composite marginal likelihood* as a means to estimate the branch length parameters.

We index transition probability matrices by DAG edges $t \rightarrow s$. To avoid deep subscripting, we will use a function-type representation ${\textbf{P}}({\varvec{\theta }}(t \rightarrow s))$, where ${\varvec{\theta }}$ is the vector of branch lengths indexed by branch $t \rightarrow s$. These transition probabilities are completely defined by the branch length vector ${\varvec{\theta }}$, because we assume no parameters of the substitution model other than branch lengths. With this assignment of branch lengths to edges, each rooted topology has a unique assignment of branch lengths and thus a well-defined likelihood.

DAG nodes can be unambigiously labeled with their subsplits, and so we will treat DAG nodes and subsplits interchangeably. We associate *p*- and *r*-PLVs to each node of the subsplit DAG. The *p*-PLVs are computed in the *rootward traversal* whereas the *r*-PLVs are computed in the *leafward traversal*. We describe details of the two passes in the subsequent sections.

We will extend the notation in the two-pass algorithm in Eqs. (3) and (4). Previously, we defined ${\textbf{p}}$ and $\textbf{r}$ as partial likelihoods of the observed sequences as functions of (partial) topologies for a given node *v*: ${\textbf{p}}(\tau ^{\downarrow }_v)$ and $\textbf{r}(\tau ^{\uparrow }_v)$. Below we will extend this notation by defining ${\textbf{p}}(t)$ as a partial likelihood vector for a subsplit *t*, which is obtained via marginalization of possible topologies involving the subsplit *t* using the partial likelihoods ${\textbf{p}}(\tau ^{\downarrow }_t)$. Similarly, we will define a $\textbf{r}((s,{\underline{Z}}))$ (where $(s,{\underline{Z}})$ will be either $\acute{s}$ or $\grave{s}$) using a similar marginalization involving $\textbf{r}(\tau ^{\uparrow }_{s,{\underline{Z}}})$. In order to compute these terms we introduce intermediate sums $\breve{{\textbf{p}}}(\acute{s})$, $\breve{{\textbf{p}}}(\grave{s})$, and $\breve{{\textbf{r}}}(s)$.

### Rootward traversal

In the rootward traversal we assume that we visit a node after visiting all of its descendants, such as via a postorder traversal.

Given a subsplit $t = \{A,B\}$, let the *leafward topologies of* *t*, denoted ${\mathcal {T}}_{{\text {leaf}}}(t)$, be the set of rooted topologies on the taxon set $A \cup B$ with *t* as the bipartition at the root of the topology. As above, we will use the notation $\tau ^{\downarrow }$ to emphasize that these are *not* topologies on the entire taxon set *X*, but only specify structure for a topology “below” a subsplit. We define the partial likelihood vector ${\textbf{p}}(t)$ of a non-leaf subsplit *t* as

9 $$\begin{aligned} {\textbf{p}}(t):= \sum _{\tau ^{\downarrow }_t \in {\mathcal {T}}_{{\text {leaf}}}(t)} {\textbf{p}}(\tau ^{\downarrow }_t) \, {\mathbb {P}}(\tau ^{\downarrow }_t \mid t), \end{aligned}$$

where ${\textbf{p}}(\tau ^{\downarrow }_t)$ is the partial likelihood vector as defined by (3) at the root of a topology $\tau ^{\downarrow }_t$ on *U*(*t*) and ${\mathbb {P}}(\tau ^{\downarrow }_t \mid t)$ is the prior probability of $\tau ^{\downarrow }_t$ given *t* as described above. If *t* is a leaf subsplit $(\{x\}, \emptyset )$, then ${\textbf{p}}(t)$ is the tip partial likelihood vector for the taxon *x*, i.e., the vector with one entry corresponding to the observed nucleotide base set to 1 and the remaining entries set to 0.

We can calculate ${\textbf{p}}(t)$ via a dynamic program generalizing the single-tree case:

#### Lemma 1

Given a subsplit *t*,

10 $$\begin{aligned} {\textbf{p}}(t) = \left( \sum _{s_1 \leftarrow \acute{t}} {\textbf{P}}({\varvec{\theta }}(\acute{t} \rightarrow s_1)) {\textbf{p}}(s_1) \, {\mathbb {P}}(s_1 \mid \acute{t}) \right) \circ \left( \sum _{s_2 \leftarrow \grave{t}} {\textbf{P}}({\varvec{\theta }}(\grave{t} \rightarrow s_2)) {\textbf{p}}(s_2) \, {\mathbb {P}}(s_2 \mid \grave{t}) \right) . \end{aligned}$$

Here and below we use $s_1 \leftarrow \acute{t}$ as an abbreviation for $\{s_1: \acute{t} \rightarrow s_1\}$, i.e. the set of subsplits $s_1$ of ${\mathcal {D}}$ that are direct descendants of the left subsplit-clade of *t*.

#### Proof

We start with the right-hand side of Eq. (10) and show equality with the left-hand side. For each $j = 1, 2$, substitute in

$$\begin{aligned} {\textbf{p}}(s_j) = \sum _{\tau ^{\downarrow }_j \in {\mathcal {T}}_{{\text {leaf}}}(s_j)} {\textbf{p}}(\tau ^{\downarrow }_j) \, {\mathbb {P}}(\tau ^{\downarrow }_j \mid s_j). \end{aligned}$$

By pulling out sums and rearranging the order of terms, we have the sum of the following quantity over subsplits $s_1 \leftarrow \acute{t}$ and $s_2 \leftarrow \grave{t}$:

$$\begin{aligned} \sum _{\begin{array}{c} \tau ^{\downarrow }_1 \in {\mathcal {T}}_{{\text {leaf}}}(s_1) \\ \tau ^{\downarrow }_2 \in {\mathcal {T}}_{{\text {leaf}}}(s_2) \end{array}} \! \! \! \! \left( {\textbf{P}}({\varvec{\theta }}(\acute{t} \rightarrow s_1)) {\textbf{p}}(\tau ^{\downarrow }_1) \right) \circ \left( {\textbf{P}}({\varvec{\theta }}(\grave{t} \rightarrow s_2)) {\textbf{p}}(\tau ^{\downarrow }_2) \right) {\mathbb {P}}(s_1 \mid \acute{t}) \, {\mathbb {P}}(\tau ^{\downarrow }_1 | s_1) {\mathbb {P}}(s_2 \mid \grave{t}) \, {\mathbb {P}}(\tau ^{\downarrow }_2 | s_2). \end{aligned}$$

We define $\tau ^{\downarrow }_t$ to be the topology built by joining $\tau ^{\downarrow }_1$ and $\tau ^{\downarrow }_2$ using *t*, so that

$$\begin{aligned} {\textbf{p}}(\tau ^{\downarrow }_t) = \left( {\textbf{P}}({\varvec{\theta }}(\acute{t} \rightarrow s_1)) {\textbf{p}}(\tau ^{\downarrow }_1) \right) \circ \left( {\textbf{P}}({\varvec{\theta }}(\grave{t} \rightarrow s_2)) {\textbf{p}}(\tau ^{\downarrow }_2) \right) \end{aligned}$$

by Eq. (5), and

$$\begin{aligned} {\mathbb {P}}(\tau ^{\downarrow }_t \mid t) = {\mathbb {P}}(s_1 | \acute{t}) \, {\mathbb {P}}(\tau ^{\downarrow }_1 | s_1) \, {\mathbb {P}}(s_2 | \grave{t}) \, {\mathbb {P}}(\tau ^{\downarrow }_2 | s_2) \end{aligned}$$

by Eq. (8). Under this construction for $\tau ^{\downarrow }_t$, the sums over subsplits $s_1 \leftarrow \acute{t}$ and $s_2 \leftarrow \grave{t}$ combined with the sums over the leafward topologies of $s_1$ and $s_2$ are equivalent to a single sum over the leafward topologies of *t*. This concludes the proof by the definition of ${\textbf{p}}(t)$. $\square$

### Rootward topologies

We have equivalent notions of partial likelihood for leafward traversals. Although conceptually quite similar, we will require additional definitions and notation.

First, we need a notion of a rooted topology where the structure of the tree is unspecified for a subset of the taxa that appear together in the tree; this will generalize the notion of $\tau ^{\uparrow }_v$ defined above. We formalize this by expressing a partially-specified topology as the set of subsplits it contains. For $Z \subset X$, define a *Z*-*unspecified topology* as a set of subsplits of the form $C \setminus D$, where *C* is the subsplit representation of a topology $\tau$ on *X* with *Z* as a clade, and *D* is the subsplit representation of the sub-topology of $\tau$ on *Z*. Furthermore, there is a natural definition of the probability of such a partially-specified topology as the product of the corresponding set of probabilities in Eq. (7).

**Fig. 4:**
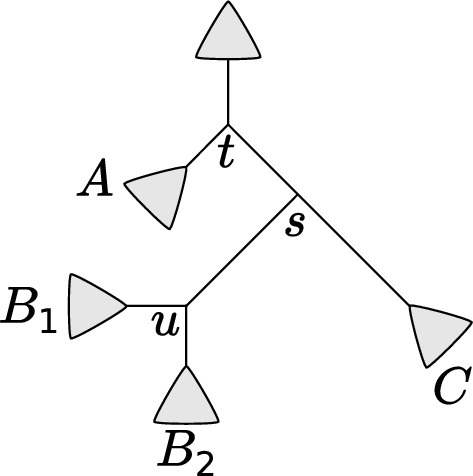
The setting for dynamic computation during the leafward pass. Assume $A< B_1< B_2 < C$ in the lexicographic ordering. Here $u = \{B_1, B_2\}$, $s = \{B_1 \cup B_2, C\}$, and $t = \{A, B_1 \cup B_2 \cup C\}$. We will use $\tau ^{\uparrow }_{\grave{t}}$ to represent the partially-specified topology with all subsplits in clade *A*, *t*, and those towards the root from *t*. We will use $\tau ^{\downarrow }_u$ to represent the topology leafward of *u* (including *u* itself)

Given a subsplit-clade $(s,{\underline{Z}})$, let the *rootward topologies of* $(s,{\underline{Z}})$, denoted by ${\mathcal {T}}_{{\text {root}}}((s,{\underline{Z}}))$, be the set of *Z*-unspecified topologies on *X* containing *s*. We emphasize that such a topology does specify topological structure for the non-*Z* side of the subsplit (often called the “sister clade” of *Z*). For example in Fig. 4, ${\mathcal {T}}_{{\text {root}}}(\grave{s})$ would be all of the *C*-unspecified topologies in the subsplit DAG containing *s* (such trees do specify structure for the sister clade of *C*, which in this case is the union of $B_1$ and $B_2$).

We can combine a leafward topology and a rootward topology and evaluate its probability dynamically using subsplits analogous to (8). Say we have subsplits *t*, *s*, and *u* such that that $\acute{s} \rightarrow u$ and $\grave{t} \rightarrow s$ (Fig. 4). Assume we are given $\tau ^{\uparrow }_{\grave{t}} \in {\mathcal {T}}_{{\text {root}}}(\grave{t})$ and $\tau ^{\downarrow }_u \in {\mathcal {T}}_{{\text {leaf}}}(u)$, and form $\tau ^{\uparrow }_{\grave{s}} \in {\mathcal {T}}_{{\text {root}}}(\grave{s})$ by joining together $\tau ^{\uparrow }_{\grave{t}}$ and $\tau ^{\downarrow }_u$ using *s*. By definition of the structures, we have

11 $$\begin{aligned} {\mathbb {P}}(\tau ^{\uparrow }_{\grave{s}}) = {\mathbb {P}}(\tau ^{\uparrow }_{\grave{t}}) \, {\mathbb {P}}(s \mid \grave{t}) \, {\mathbb {P}}(u \mid \acute{s}) \, {\mathbb {P}}(\tau ^{\downarrow }_{u} \mid u). \end{aligned}$$

The equivalent equation holds (using a suitable definition of *u*) when replacing $\grave{t}$ with $\acute{t}$, or $\grave{s}$ with $\acute{s}$. This is analogous to (8), but there are important differences. For example, (8) gives a probability ${\mathbb {P}}(\tau |t)$ conditioned on a subsplit *t*, but no such conditioning is present for (11).

### Leafward traversal

We now use our PLVs ${\textbf{p}}$ marginalizing over leafward trees to build the PLVs $\textbf{r}$ marginalizing over rootward trees. This happens in a reverse post-order traversal, which we call the “leafward traversal”.

First we need a version of the $\textbf{r}$ vector that is defined for an element of $\tau ^{\uparrow }_{s,{\underline{Z}}} \in {\mathcal {T}}_{{\text {root}}}((s,{\underline{Z}}))$:

12 $$\begin{aligned} {\textbf{r}{(\tau ^{\uparrow }_{s,{\underline{Z}}})}}_i:= {\mathbb {P}}({\textbf{Y}}_{X \setminus Z},Y_{s} = i \mid \tau ^{\uparrow }_{s,{\underline{Z}}}) \end{aligned}$$

where ${\textbf{Y}}_{X \setminus Z}$ is the data for all taxa outside of *Z* and $Y_{s}$ is the state of the node corresponding to subsplit *s* in the tree. This is equivalent to the definition (4) of $\textbf{r}$ for any tree containing $\tau ^{\uparrow }_{s,{\underline{Z}}}$ such that *v* is the root node of the subtree containing all the taxa of *Z*.

We formalize our topology-marginal version of $\textbf{r}$ for subsplit-clades $(s,{\underline{Z}})$:

13 $$\begin{aligned} \textbf{r}((s,{\underline{Z}})):= \sum _{\tau ^{\uparrow }_{s,{\underline{Z}}} \in {\mathcal {T}}_{{\text {root}}}((s,{\underline{Z}}))} \textbf{r}(\tau ^{\uparrow }_{s,{\underline{Z}}}) \, {\mathbb {P}}(\tau ^{\uparrow }_{s,{\underline{Z}}}). \end{aligned}$$

The summand is the joint probability of observing $\tau ^{\uparrow }_{s,{\underline{Z}}}$ in the DAG and ${\textbf{Y}}_{X \setminus Z}$ (all of the sequences other than those in *Z*). There are important differences between this and (9): we use an unconditional ${\mathbb {P}}(\tau ^{\uparrow }_{s,{\underline{Z}}})$ of a partially specified topology $\tau ^{\uparrow }_{s,{\underline{Z}}}$ where before we used a conditional ${\mathbb {P}}(\tau ^{\downarrow }_t|t)$ on a fully specified topology for the subset of taxa in *t*.

Rewriting (6) in DAG notation, we have

14 $$\begin{aligned} \textbf{r}(\tau ^{\uparrow }_{\grave{s}}) = \left( {\textbf{P}}({\varvec{\theta }}(\acute{s} \rightarrow u)) {\textbf{p}}(\tau ^{\downarrow }_u) \right) \circ \left( {\textbf{P}}^\top ({\varvec{\theta }}(\grave{t} \rightarrow s)) \textbf{r}(\tau ^{\uparrow }_{\grave{t}}) \right) . \end{aligned}$$

With this we can derive the leafward-traversal version of (10). Recall that $\textbf{r}(\tau ^{\uparrow }_{\grave{s}})$ is of the form $\textbf{r}((s,{\underline{Z}}))$, so the goal is to show that (13) decomposes into the element-wise product ($\circ$) of two terms.

#### Lemma 2

   

15 $$\begin{aligned} \begin{aligned} \textbf{r}(\grave{s})&= \left( \sum _{u: \acute{s} \rightarrow u} {\textbf{P}}({\varvec{\theta }}(\acute{s} \rightarrow u)) {\textbf{p}}(u) \, {\mathbb {P}}(u \mid \acute{s}) \right) \\&\quad \circ \left( \sum _{\grave{t}: \grave{t} \rightarrow s} {\textbf{P}}^\top ({\varvec{\theta }}(\grave{t} \rightarrow s)) \textbf{r}(\grave{t}) \, {\mathbb {P}}(s \mid \grave{t}) \ + \sum _{\acute{t}: \acute{t} \rightarrow s} {\textbf{P}}^\top ({\varvec{\theta }}(\acute{t} \rightarrow s)) \textbf{r}(\acute{t}) \, {\mathbb {P}}(s \mid \acute{t}) \right) . \end{aligned} \end{aligned}$$

The same holds true exchanging $\grave{s}$ and $\acute{s}$.

We emphasize that this is a strict generalization of Felsenstein’s pruning algorithm, because in the single-tree case the ${\mathbb {P}}$ are indicator functions so the sum collapses to a single term.

#### Proof

We start with the right-hand side and show equality with the left-hand side. Because $\circ$ is linear, we can split (15) into the sum of two terms. We focus on the product of sums indexed by *u* and $\grave{t}$, and substitute in

$$\begin{aligned} {\textbf{p}}(u) = \sum _{\tau ^{\downarrow }_u \in {\mathcal {T}}_{{\text {leaf}}}(u)} {\textbf{p}}(\tau ^{\downarrow }_u) \, {\mathbb {P}}(\tau ^{\downarrow }_u|u) \end{aligned}$$

and

$$\begin{aligned} \textbf{r}(\grave{t}) = \sum _{\tau ^{\uparrow }_{\grave{t}} \in {\mathcal {T}}_{{\text {root}}}(\grave{t})} \textbf{r}(\tau ^{\uparrow }_{\grave{t}}) \, {\mathbb {P}}(\tau ^{\uparrow }_{\grave{t}}). \end{aligned}$$

Upon pulling sums out and rearranging the order of terms, we have

$$\begin{aligned} \sum _{\begin{array}{c} u: \acute{s} \rightarrow u \\ \grave{t}: \grave{t} \rightarrow s \end{array}} \sum _{\begin{array}{c} \tau ^{\downarrow }_u \in {\mathcal {T}}_{{\text {leaf}}}(u) \\ \tau ^{\uparrow }_{\grave{t}} \in {\mathcal {T}}_{{\text {root}}}(\grave{t}) \end{array}} & \! \! \! \! \left( {\textbf{P}}({\varvec{\theta }}(\acute{s} \rightarrow u)) {\textbf{p}}(\tau ^{\downarrow }_u) \right) \\ & \circ \left( {\textbf{P}}^\top ({\varvec{\theta }}(\grave{t} \rightarrow s)) \textbf{r}(\tau ^{\uparrow }_{\grave{t}}) \right) \, \\ \ & {\mathbb {P}}(\tau ^{\uparrow }_{\grave{t}}) \, {\mathbb {P}}(s \mid \grave{t}) \, {\mathbb {P}}(u \mid \acute{s}) \, {\mathbb {P}}(\tau ^{\downarrow }_u|u). \end{aligned}$$

Similar to the proof of Lemma 1, there is a 1-to-1 correspondence between such $(\tau ^{\uparrow }_{\grave{t}}, \tau ^{\downarrow }_u)$ pairs and topologies $\tau ^{\uparrow }_{\grave{s}} \in {\mathcal {T}}_{{\text {root}}}(\grave{s})$, when restricted to those topologies $\tau ^{\uparrow }_{\grave{s}}$ where $\grave{s}$ descends as the right subsplit of its parent: if we are given $(\tau ^{\uparrow }_{\grave{t}}, \tau ^{\downarrow }_u)$ we simply join them together using *s* to make a $\tau ^{\uparrow }_{\grave{s}} \in {\mathcal {T}}_{{\text {root}}}(\grave{s})$. This concludes the proof for the first term by (11), (14), and the definition of $\textbf{r}(\grave{s})$. The second term, using $\acute{t}$, follows in exactly the same way, as does the statement when exchanging $\acute{s}$ and $\grave{s}$. Note that we need both of these terms because of where the subsplit *s* appears relative to the tree above it: it could appear as either a left or a right subsplit of the parent split. $\square$

### Implementing efficient computation

We now introduce additional notation to represent and store intermediate computations.

16 $$\begin{aligned} \breve{{\textbf{p}}}(\grave{s})&:= \sum _{u: \grave{s} \rightarrow u} {\textbf{P}}({\varvec{\theta }}(\grave{s} \rightarrow u)) {\textbf{p}}(u) \, {\mathbb {P}}(u \mid \grave{s}) , \end{aligned}$$

17 $$\begin{aligned} \breve{{\textbf{p}}}(\acute{s})&:= \sum _{u: \acute{s} \rightarrow u} {\textbf{P}}({\varvec{\theta }}(\acute{s} \rightarrow u)) {\textbf{p}}(u) \, {\mathbb {P}}(u \mid \acute{s}) , \end{aligned}$$

18 $$\begin{aligned} \breve{{\textbf{r}}}(s)&:= \sum _{\grave{t}: \grave{t} \rightarrow s} {\textbf{P}}^\top ({\varvec{\theta }}(\grave{t} \rightarrow s)) \textbf{r}(\grave{t}) \, {\mathbb {P}}(s \mid \grave{t}) + \sum _{\acute{t}: \acute{t} \rightarrow s} {\textbf{P}}^\top ({\varvec{\theta }}(\acute{t} \rightarrow s)) \textbf{r}(\acute{t}) \, {\mathbb {P}}(s \mid \acute{t}). \end{aligned}$$

With these definitions, (10) and  (15) become

19 $$\begin{aligned} {\textbf{p}}(s)&= \breve{{\textbf{p}}}(\acute{s}) \circ \breve{{\textbf{p}}}(\grave{s}), \end{aligned}$$

20 $$\begin{aligned} \textbf{r}(\grave{s})&= \breve{{\textbf{p}}}(\acute{s}) \circ \breve{{\textbf{r}}}(s), \end{aligned}$$

21 $$\begin{aligned} \textbf{r}(\acute{s})&= \breve{{\textbf{p}}}(\grave{s}) \circ \breve{{\textbf{r}}}(s) . \end{aligned}$$

**Fig. 5:**
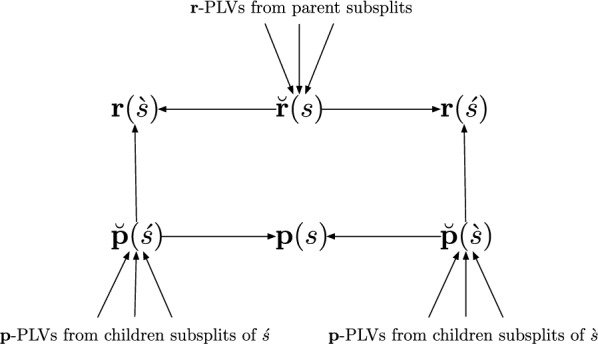
Dependency graph of partial likelihood vectors associated with a subsplit *s*, where $x\,\rightarrow \, y$ means that *y* depends on *x*

We use the dependency graph to calculate the ensemble of partial likelihood vectors (Fig. 5) associated with each subsplit *s*. We perform the *rootward traversal* to compute $\breve{{\textbf{p}}}(\acute{s})$ and $\breve{{\textbf{p}}}(\grave{s})$ using ${\textbf{p}}(u)$ for $u: \acute{s} \rightarrow u$ and $u: \grave{s} \rightarrow u$, giving ${\textbf{p}}(s)$ by (16), (17), and (19). Given these vectors our second pass, the *leafward traversal*, computes $\breve{{\textbf{r}}}(s)$ via (18), (20), and (21).

### The per-edge marginal likelihood

For a given edge $t \rightarrow s$, define the *per edge marginal likelihood* to be

22 $$\begin{aligned} \ell (t \rightarrow s; {\varvec{\theta }}):= \sum _{\tau \ni (t \rightarrow s)} {\mathbb {P}}({\textbf{Y}}\mid \tau , {\varvec{\theta }}) \, {\mathbb {P}}(\tau \mid t \rightarrow s). \end{aligned}$$

Here $t \rightarrow s$ is interpreted as $\grave{t}\rightarrow s$ or $\acute{t}\rightarrow s$, whichever is correct (as above). We are introducing a new notion, ${\mathbb {P}}(\tau \mid t \rightarrow s)$, which is a normalized probability restricted to the trees that contain an edge $t \rightarrow s$ of the DAG:

$$\begin{aligned} {\mathbb {P}}(\tau \mid t \rightarrow s):= \frac{{\mathbb {P}}(\tau ) \, \mathbbm {1}[t \rightarrow s \in \tau ]}{\sum _{\tau ' \in {\mathcal {D}}} {\mathbb {P}}(\tau ') \, \mathbbm {1}[t \rightarrow s \in \tau ']}. \end{aligned}$$

The numerator defines the joint probability of observing a tree and an edge,

$$\begin{aligned} {\mathbb {P}}(\tau , t \rightarrow s):= {\mathbb {P}}(\tau ) \, \mathbbm {1}[t \rightarrow s \in \tau ]. \end{aligned}$$

The denominator is the unconditional probability of sampling a tree containing an edge $t \rightarrow s$:

23 $$\begin{aligned} {\mathbb {P}}(t \rightarrow s):= \sum _{\tau \in {\mathcal {D}}} {\mathbb {P}}(\tau ) \, \mathbbm {1}[t \rightarrow s \in \tau ], \end{aligned}$$

note that we sum over $\tau$ so this quantity is independent of the tree topologies. Now, (23) can be expressed as

24 $$\begin{aligned} {\mathbb {P}}(t \rightarrow s) = {\mathbb {P}}(t) \cdot {\mathbb {P}}(s | t), \end{aligned}$$

where ${\mathbb {P}}(t)$ is the unconditional probability of sampling a tree containing a subsplit *t*.

We now show how to calculate $\ell (t \rightarrow s; {\varvec{\theta }})$ efficiently using the components already described. A key part is that we can expand $P({\textbf{Y}}| \tau , {\varvec{\theta }})$ over any edge $t \rightarrow s \in \tau$ as per the usual Felsenstein pruning algorithm by

25 $$\begin{aligned} {\mathbb {P}}({\textbf{Y}}| \tau , {\varvec{\theta }}) = \textbf{r}(\tau ^{\uparrow }_t)^\top {\textbf{P}}({\varvec{\theta }}(t \rightarrow s)) {\textbf{p}}(\tau ^{\downarrow }_s); \end{aligned}$$

marginalization leads to the following lemma.

#### Lemma 3

   

26 $$\begin{aligned} \ell (\grave{t} \rightarrow s; {\varvec{\theta }}) = \textbf{r}(\grave{t})^\top {\textbf{P}}({\varvec{\theta }}(\grave{t} \rightarrow s)) {\textbf{p}}(s) \, / \, {\mathbb {P}}(\grave{t}). \end{aligned}$$

The same holds true exchanging $\grave{t}$ for $\acute{t}$.

#### Proof

First recall that for any $\tau$ containing $\grave{t} \rightarrow s$,

$$\begin{aligned} {\mathbb {P}}(\tau ) = {\mathbb {P}}(\tau ^{\uparrow }_{\grave{t}}) \, {\mathbb {P}}(s \mid \grave{t}) \, {\mathbb {P}}(\tau ^{\downarrow }_s | s) \end{aligned}$$

where $\tau ^{\uparrow }_{\grave{t}}$ is the *U*(*s*)-unspecified topology rootward of *t*, and $\tau ^{\downarrow }_s$ is the topology on *U*(*s*) leafward of *s*. Thus

$$\begin{aligned} {\mathbb {P}}(\tau \mid \grave{t} \rightarrow s) = \frac{{\mathbb {P}}(\tau , \grave{t} \rightarrow s)}{{\mathbb {P}}(\grave{t}) {\mathbb {P}}(s | \grave{t})} = \frac{{\mathbb {P}}(\tau ^{\uparrow }_{\grave{t}}) {\mathbb {P}}(s | \grave{t}) {\mathbb {P}}(\tau ^{\downarrow }_s | s)}{{\mathbb {P}}(\grave{t}) {\mathbb {P}}(s | \grave{t})} = \frac{{\mathbb {P}}(\tau ^{\uparrow }_{\grave{t}}) {\mathbb {P}}(\tau ^{\downarrow }_s | s)}{{\mathbb {P}}(\grave{t})}, \end{aligned}$$

where ${\mathbb {P}}(\tau , \grave{t} \rightarrow s)$ is the joint probability of observing a tree $\tau$ and having it contain the edge $\grave{t} \rightarrow s$. Using this identity and Equation (25) to expand (22), we have

$$\begin{aligned}&\sum _{\tau \ni (\grave{t} \rightarrow s)} \textbf{r}(\tau ^{\uparrow }_{\grave{t}})^\top {\textbf{P}}({\varvec{\theta }}(\grave{t} \rightarrow s)) {\textbf{p}}(\tau ^{\downarrow }_s) \, {\mathbb {P}}(\tau \mid \grave{t} \rightarrow s) \\&\quad = \sum _{\begin{array}{c} \tau ^{\uparrow }_{\grave{t}} \in {\mathcal {T}}_{{\text {root}}}(\grave{t}) \\ \tau ^{\downarrow }_s \in {\mathcal {T}}_{{\text {leaf}}}(s) \end{array}} \textbf{r}(\tau ^{\uparrow }_{\grave{t}})^\top {\textbf{P}}({\varvec{\theta }}(\grave{t} \rightarrow s)) {\textbf{p}}(\tau ^{\downarrow }_s) \, {\mathbb {P}}(\tau ^{\uparrow }_{\grave{t}}) \, {\mathbb {P}}(\tau ^{\downarrow }_s \mid s) \, / \, {\mathbb {P}}(\grave{t}) \\&\quad = \left( \sum _{\tau ^{\uparrow }_{\grave{t}} \in {\mathcal {T}}_{{\text {root}}}(\grave{t})} \textbf{r}(\tau ^{\uparrow }_{\grave{t}}) \, {\mathbb {P}}(\tau ^{\uparrow }_{\grave{t}})\right) ^\top {\textbf{P}}({\varvec{\theta }}(\grave{t} \rightarrow s)) \left( \sum _{\tau ^{\downarrow }_s \in {\mathcal {T}}_{{\text {leaf}}}(s)} {\textbf{p}}(\tau ^{\downarrow }_s) \, {\mathbb {P}}(\tau ^{\downarrow }_s|s)\right) \, \Bigg / \, {\mathbb {P}}(\grave{t}) \\&\quad = \textbf{r}(\grave{t})^\top {\textbf{P}}({\varvec{\theta }}(\grave{t} \rightarrow s)) {\textbf{p}}(s) \, / \, {\mathbb {P}}(\grave{t}). \end{aligned}$$

$$\square$$

### Composite-like marginal likelihood

So far we have described computations for a single site and dropped the site from the notation. At this point we shift to notation that is explicit about the site under consideration.

As described at the beginning, assume we have *M* sites, which are indexed by $m \in \{1, \ldots , M\}$. Our work so far enables efficient computation of

$$\begin{aligned} \ell ^m({\varvec{\theta }}):= {\mathbb {P}}({\textbf{Y}}^m \mid {\varvec{\theta }}) = \sum _{\tau \in {\mathcal {D}}} {\mathbb {P}}({\textbf{Y}}^m \mid \tau , {\varvec{\theta }}) {\mathbb {P}}(\tau ). \end{aligned}$$

Namely, we can evaluate the exact marginal likelihood for each site by summing over the rootsplits *t* for fixed values of ${\varvec{\theta }}$:

$$\begin{aligned} \ell ^m({\varvec{\theta }})&= \sum _{\tau \in {\mathcal {D}}} {\mathbb {P}}({\textbf{Y}}^m \mid \tau , {\varvec{\theta }}) \, {\mathbb {P}}(\tau ) \\&= \sum _{\text {rootsplits } t} \sum _{\tau ^{\downarrow }_t \in {\mathcal {T}}_{{\text {leaf}}}(t)} {\varvec{\pi }}^\top {\textbf{p}}^m(\tau ^{\downarrow }_t) \, {\mathbb {P}}(t) \, {\mathbb {P}}(\tau ^{\downarrow }_t \mid t) \\&= \sum _{\text {rootsplits } t} {\mathbb {P}}(t) \, {\varvec{\pi }}^\top {\textbf{p}}^m(t). \end{aligned}$$

Here ${\varvec{\pi }}$ is the distribution at the root and ${\textbf{p}}^m$ is the *p*-PLV for the *m*-th site, which was simply ${\textbf{p}}$ in the previous section. Similarly, we use $\textbf{r}^m$ for the *r*-PLV at the *m*-th site.

One approach is to combine the per-site marginal likelihood to form a composite-like objective,

27 $$\begin{aligned} \ell ({\varvec{\theta }}):= \prod _{m=1}^M \ell ^m({\varvec{\theta }}). \end{aligned}$$

In fact, we define a related notion of *per-edge composite likelihood*, and use it as an objective function when optimizing the branch length parameters associated with each edge $t \rightarrow s$. This per-edge composite likelihood is defined as the product of the per-edge marginal likelihoods over the sites,

28 $$\begin{aligned} \ell (t \rightarrow s; {\varvec{\theta }}):= \prod _{m} \ell ^m(t \rightarrow s; {\varvec{\theta }}). \end{aligned}$$

We can optimize the per edge composite marginal likelihood over branch lengths via a gradient-free method such as Brent optimization [20] since evaluating (28) is fast given the PLVs using Lemma 3 (for numerical stability, we instead optimize $\log \ell (t \rightarrow s; {\varvec{\theta }})$). Next we note how gradient-based optimization is also possible.

#### Gradient-based optimization

Let $\partial _{t \rightarrow s}$ be the partial derivative of the component of the branch length vector ${\varvec{\theta }}$ corresponding to $t \rightarrow s$. Thus

$$\begin{aligned} \partial _{t \rightarrow s} \log \ell (t \rightarrow s; {\varvec{\theta }}) = \sum _{m=1}^{M} \frac{\partial _{t \rightarrow s} \, \ell ^m(t \rightarrow s; {\varvec{\theta }})}{\ell ^m(t \rightarrow s; {\varvec{\theta }})}. \end{aligned}$$

By Lemma 3, we have,

$$\begin{aligned} \partial _{t \rightarrow s} \ell ^m(t \rightarrow s; {\varvec{\theta }})&= \frac{1}{{\mathbb {P}}(t)} \, (\textbf{r}^m(t))^\top (\partial _{t \rightarrow s} {\textbf{P}}({\varvec{\theta }}(t \rightarrow s))) {\textbf{p}}^m(s). \end{aligned}$$

Returning to the full likelihood optimization, we have

$$\begin{aligned} \partial _{t \rightarrow s} \log \ell ({\varvec{\theta }})&= \sum _{m=1}^{M} \frac{1}{\ell ^m({\varvec{\theta }})} \left[ \partial _{t \rightarrow s} \, \ell ^m({\varvec{\theta }}) \right] \\&= \frac{{\mathbb {P}}(t\rightarrow s)}{{\mathbb {P}}(t)} \sum _{m=1}^{M} \frac{1}{\ell ^m({\varvec{\theta }})} \left[ (\textbf{r}^m(t))^\top (\partial _{t \rightarrow s} {\textbf{P}}({\varvec{\theta }}(t \rightarrow s))) {\textbf{p}}^m(s) \right] . \end{aligned}$$

### Optimization in analogy with the single tree case

In the case of a single tree, it is typical for maximum-likelihood phylogenetic algorithms to proceed from edge to edge of the tree, optimizing the branch length for each edge. This is made efficient by the two-pass algorithm on the tree, where partial likelihood vectors on the tree can be calculated and then used for a constant-time function evaluation in the inner optimization loop. By optimizing the likelihood as parameterized by a single branch, they optimize the likelihood of data given the entire tree.

The setting for the DAG is related but somewhat different. We also have partial likelihood vectors, which can be calculated via a dynamic program, enabling efficient local inference. However, we do not have the guarantee that sequentially maximizing the per-edge marginal likelihood (22) will maximize the full composite likelihood (27). This is because improving the per-edge marginal likelihood for a given edge may decrease the per-edge marginal likelihood for another. In fact, this does occur in practice. Nevertheless, we have found that this algorithm works well for our purposes.

### Optimization schedule

We begin by describing a slightly simplified version of the algorithm, and will then describe the full version. Given an initial branch length ${\varvec{\theta }}$, we perform a rootward traversal to populate all of the ${\textbf{p}}$-PLVs followed by a leafward traversal to populate $\textbf{r}$-PLVs. We then perform a depth-first traversal as follows to optimize the branch lengths while keeping track of visited nodes with a set *S* so as to not re-visit nodes more than once. When we visit a given node *t*, we begin by updating $\breve{{\textbf{r}}}(t)$ as the marginal of the $\textbf{r}$-PLVs of the parent subsplits. This is an important step as each subsplit node *s* updates $\breve{{\textbf{r}}}(s)$ and passes down the results to its children, so that optimization for a edge has up-to-date information from other parts of the graph. When a non-trivial subsplit is visited, we descend into the left subsplit-clade followed by the right subsplit-clade (or vice versa) to keep it consistent. Specifically, we update $\textbf{r}(\acute{t})$ using $\breve{{\textbf{r}}}(t)$ and $\breve{{\textbf{p}}}(\grave{t})$ computed from the previous optimization iteration. Then for each $\acute{t} \rightarrow s \in {\mathcal {D}}$, we optimize all branches below *s* and update ${\textbf{p}}(s)$. Upon returning from the recursion on child *s*, we optimize ${\varvec{\theta }}(\acute{t} \rightarrow s)$ by maximizing $\textbf{r}(\acute{t})^\top {\textbf{P}}(\theta ) {\textbf{p}}(s)$. We accumulate $\breve{{\textbf{p}}}(\acute{t})$ using the optimized ${\varvec{\theta }}(\acute{t} \rightarrow s)$ for each child *s*. Once the recursion on left subsplit-clade completes, we repeat the same procedure for $\grave{t}$; finally, we update ${\textbf{p}}(t) = \breve{{\textbf{p}}}(\acute{t}) \circ \breve{{\textbf{p}}}(\grave{t})$. The optimization is run until convergence is reached. Pseudocode for this simplified procedure is outlined in Algorithm 1.

However, this simple version has a shortcoming in that, because of the structure of the DAG, modification of one branch length can invalidate some of the PLVs used later in the procedure. To handle this, we perform a modified depth-first traversal on a version of the DAG in which each node can be marked with a “dirty” status. If a node *s* is dirty, then we assume that ${\textbf{p}}(s)$, $\breve{{\textbf{p}}}(\acute{s})$, and $\breve{{\textbf{p}}}(\grave{s})$ are invalid. If we modify a branch length for a given edge of the subsplit DAG, then we must mark all of the ancestors of the nodes of that edge as dirty. If we require the PLV for a dirty node, then we perform a traversal down, performing updates without optimizing branch lengths, until the node is clean. Note that because we do not re-optimize branch lengths when “cleaning” a node, this cannot lead to optimization loops.

We draw parallels between our algorithm to the loopy belief propagation (LBP) [5]. Although the convergence is not guaranteed for LBP and there may be oscillations, the algorithm is known to produce accurate results through empirical studies, especially when the subgraphs have tree-like properties [21, 22]. In the experiments section, we show that the proposed parameter estimation scheme recovers branch lengths that are close to the truth.
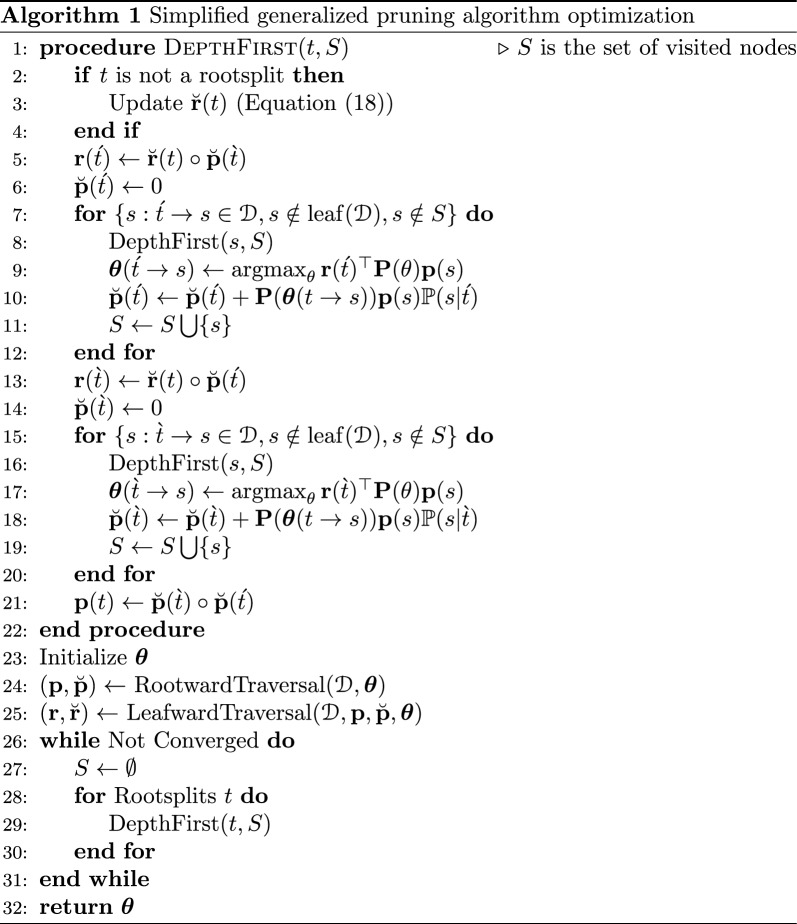


### Prior on subsplit parameters

As a prior on subsplit parameters, the simplest choice would be to set ${\mathbb {P}}(s \mid t) \propto 1$. However, this does not correspond to any previously described prior on topologies. Another option is to initialize ${\mathbb {P}}$ so as to induce a uniform prior on topologies appearing in the DAG. To that end, we must first compute *n*(*t*), the number of topologies in the DAG descending from the subsplit *t* (we use the same notation for *n* applied to a subsplit-clade). Using this we can take a prior $p_{\textrm{pr}}$ on rootsplits and edges:

29 $$\begin{aligned} {\mathbb {P}}_{{\text {prior}}}(t):= \frac{n(\acute{t}) n(\grave{t})}{n_{\textrm{total}}} \qquad {\mathbb {P}}_{{\text {prior}}}(s | (t,{\underline{Z}})):= \frac{n(s)}{n((t,{\underline{Z}}))} \end{aligned}$$

where $n_{\textrm{total}}$ is the total number of topologies in the DAG. One can see that this leads to a uniform prior on topologies by expanding an arbitrary tree into its component subsplits. Computation of $n(t), n({\tilde{t}}), n_{\textrm{total}}$ can be performed using a simple recursive algorithm.

## Implementation and experiments

We implemented the generalized pruning algorithm in our Python-interface C++ library bito (https://github.com/phylovi/bito). In this section, we test accuracy, speed, and scalability of the generalized pruning implementation on phylogenetic datasets of various sizes. Our experiments can be re-run as described in the section *Availability of data and materials* below.

### Accuracy

We want to know if optimization of the composite likelihood using the generalized pruning algorithm could give a rapid and accurate estimate of part of the phylogenetic model. To that end, we compared branch length estimates between GP and those observed in posterior samples of a standard MCMC run on empirical data sets. The data sets, which we call DS1 and DS3-DS8, are standard data sets for evaluating MCMC methods on phylogenies (e.g., [1, 6, 7, 23]). We excluded DS2 from this study because it has an almost trivial posterior distribution [23]. The data sets consist of sequences from 27 to 67 species and are fully described in [1].

**Table 1:** The Pearson correlation and mean absolute error between GP and VBPI estimates to the MCMC posterior means. VBPI outliers were defined as branch length estimates outside the 95% quantile

Dataset	Correlation			Mean absolute error		
	GP	VBPI	VBPI outliers omitted	GP	VBPI	VBPI outliers omitted
DS1	0.991	0.102	0.909	0.0009	0.0032	0.0012
DS3	0.999	0.999	0.999	0.0015	0.0007	0.0007
DS4	0.999	0.166	0.970	0.0012	0.0085	0.0019
DS5	0.990	0.930	0.920	0.0044	0.0050	0.0047
DS6	0.995	− 0.017	0.858	0.0011	0.0240	0.0013
DS7	0.999	0.999	0.999	0.0010	0.0003	0.0003
DS8	0.995	0.975	0.950	0.0015	0.0013	0.0012

We used MrBayes 3.2 [24] to obtain posterior samples to serve as the ground truth for accuracy assessments. For each data set we ran 4 chains of 1,000,000,000 generations; sampled every 1000 generations; and used the first 100,000,000 generations as a burn-in to yield 900,000 samples from the posterior. The posterior branch length estimates were obtained by rooting topologies in the posterior and calculating the average branch length across topologies for each observed DAG edge. As we are interested in the accuracy of the estimation procedure developed here, rather than construction of the DAG, we constructed the DAG using the topologies found in the MrBayes posterior samples for the experiments.

We also assessed how competitive GP is against a state-of-the-art variational Bayesian phylogenetic inference (VBPI) method [9]. VBPI postulates variational approximations of the posterior using a product of two distributions, one for the topology and the other for the branch lengths. The topology component of the variational approximation is parameterized using subsplits; by supplying the same set of topologies as an input to GP and VBPI, we can ensure fairness in evaluation. VBPI optimizes the parameters of the two distributions iteratively using stochastic gradient descent, where each iteration involves a sampling step to draw phylogenetic trees (topology and branch lengths) from the variational distributions followed by a gradient step to optimize variational parameters. Upon convergence, the fitted variational distributions have shown to approximate the posterior distribution with high accuracy.

We applied VBPI on the same DS datasets to fit the parameters of the variational distribution and compared against GP for both speed and accuracy. We ran the VBPI implementation on each data set for 200,000 iterations, with 10 trees sampled per iteration for stochastic gradient descent, and output 1,000,000 trees sampled from the converged distribution. The 1,000,000 trees were processed to give average branch lengths in the same manner as the 900,000 trees sampled from the posterior.

We compared the posterior means to the estimates output by loading the same sample of topologies into GP and VBPI to obtain branch length estimates per edge of the DAG. Additionally, we calculated the coverage of the estimates to the 95% credible intervals, measured as the percentage of DAG edge branch length estimates that fall between the 2.5 and 97.5 percentiles in the posterior samples for each dataset. To ensure reliability of the posterior means and credible intervals, we discarded all edges appearing in fewer than 10 posterior samples.

Some care was required to ensure a fair comparison to the posterior for either method. The VBPI implementation places an exponential prior on the branch lengths, while our current implementation of GP implicitly assumes a uniform prior. We thus use MrBayes posterior samples that match these prior assumptions for the comparison. For comparison of GP to MrBayes, we use posterior samples obtained with the MrBayes specifications as described earlier with the Uniform (0, 1) prior on the branch lengths. For comparison of VBPI to MrBayes, we use posterior samples obtained with those same settings, except with the Exponential (10) prior on the branch lengths. We do note that by aggregating branch length results by DAG edge, GP may have a slight inherent advantage because it optimizes branch lengths on a per-edge basis. In contrast, VBPI has a different and more complex parameterization for unrooted trees [9].

**Fig. 6:**
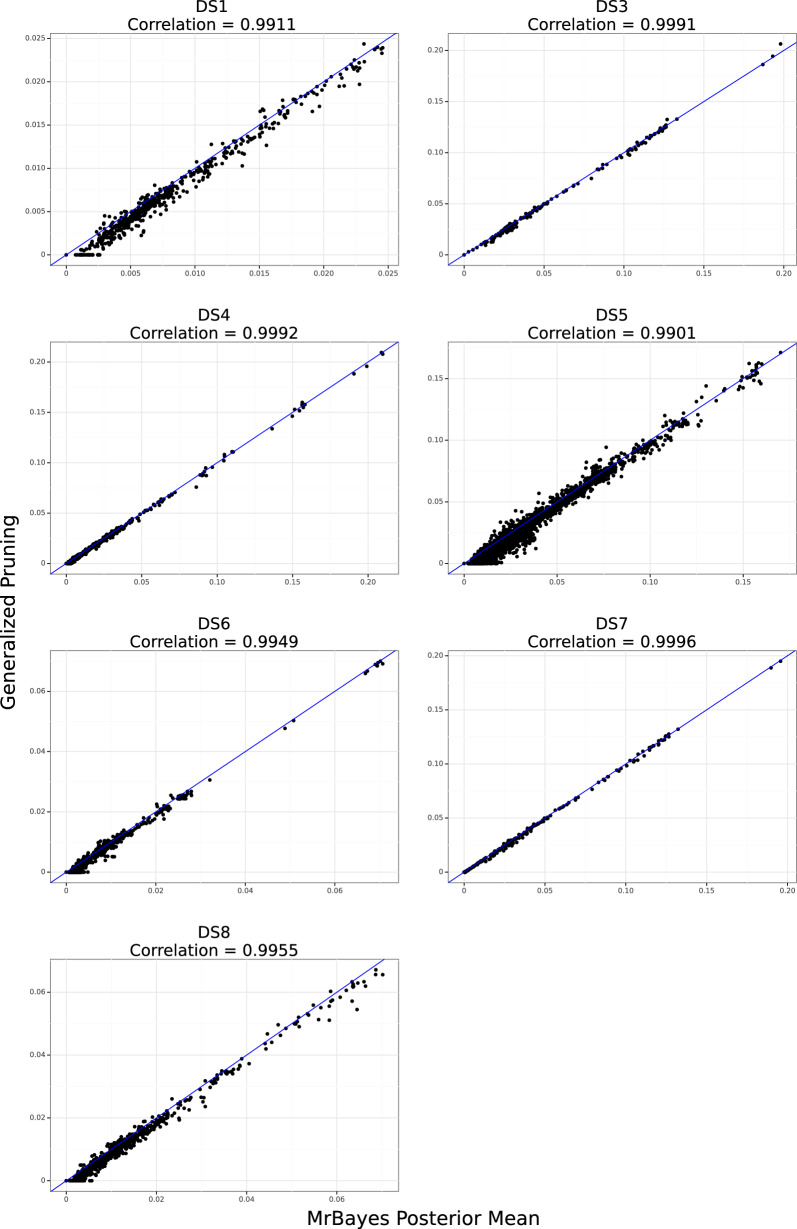
Scatter plot of GP estimates vs MrBayes posterior means on the branch lengths for DS1, DS3, DS4, DS5, DS6, DS7, and DS8 for sDAG edges that appear in at least 10 posterior samples. The MrBayes posterior was sampled with a Uniform(0, 1) prior on branch lengths

We compared the correlation and mean absolute error values between the estimates, shown in Table 1. We see that GP estimates closely align with those obtained from MCMC and offer improvements on both metrics compared to VBPI. Table 2 shows that GP and VBPI achieve similar coverage of the 95% credible interval across most datasets, with VBPI showing notably higher coverage for DS6. The scatter plots of GP vs the posterior means for each dataset are provided in Fig. 6. There is strong concordance between the GP estimates and the posterior means, from which we conclude that the composite likelihood optimization yields accurate estimates of the branch lengths. VBPI also mostly reports accurate estimates, although are some outliers for DS1, DS4, DS5, and DS6 (Additional file 1: Figure S1).

### Speed and scalability

We wanted to evaluate the speed of the generalized pruning algorithm. We should note that VBPI is written in Python and calls PyTorch, whereas GP is written in C++, which can explain some of the speed difference. However recent benchmarking [25] shows that the automatic differentiation gradients used in VBPI are within an order of magnitude of carefully optimized implementations [26].

**Table 2:** Coverage of GP and VBPI estimates in the 95% credible intervals

Dataset	GP	VBPI
DS1	0.946	0.926
DS3	0.983	1.000
DS4	0.958	0.915
DS5	0.907	0.921
DS6	0.709	0.870
DS7	0.849	0.997
DS8	0.893	0.950

**Table 3:** Timing results for executing GP and VBPI given a set of trees to build the subsplit DAG. GP runtimes are averages over 10 replicates of DAG initialization and branch length estimation

Dataset	DAG initialization (s)	GP estimation runtime (s)	VBPI runtime (s)
DS1	0.58	1.72	11, 336
DS3	0.19	0.38	15, 273
DS4	1.29	1.81	16, 867
DS5	62.11	4.90	19, 648
DS6	40.05	2.64	20, 420
DS7	1.72	0.67	25, 587
DS8	6.81	1.04	25, 725

VBPI requires significantly longer runtime to converge compared to GP as shown in Table 3, but it is worth noting that VBPI learns branch length distributions rather than single point estimates, and additionally learns subsplit probabilities. We ran GP 10 times on each of the data set to obtain average run times estimates for the initialization of the DAG and estimation of the branch lengths. We measured DAG initialization as the time needed to build the subsplit DAG from the set of unique topologies found in the very large MrBayes posterior sample. This does not include inference of the trees used to build the subsplit DAG, nor does it include processing and deduplicating the raw MrBayes output into the format needed to build the DAG.

In order to understand the opportunities for scaling generalized pruning to large data sets, we measured the actual computation time for generalized pruning on a large subsample of sequences of the Makona variant Ebola virus [27]. We ran a single-threaded implementation of generalized pruning on an Intel Xeon E5-2667 Processor running Ubuntu 18.04 with 256 GB of RAM. This sample contained 1570 sequences and the subsplit DAG was built from a set of 1,000 trees obtained from an Ultrafast Bootstrap approximation using IQ-Tree [28]. The resulting subsplit DAG contained 42,305 nodes and 92,148 edges, which required 120 GB of virtual memory (allocated via a call to Linux mmap). Building the subsplit DAG required 25 min and 6 s, while branch length estimation required 2 full DAG traversals in 21 min and 26 s. There are further opportunities for efficient computations on large data sets, such as parallelization of branch length optimization in suitably distant edges in the DAG.

### Availability of data and materials

All data, scripts, and instructions for reproducing the results presented are available at https://github.com/matsengrp/gp-benchmark-1. Readers can independently reproduce the MrBayes posterior samples, branch length estimates on the DS datasets for both VBPI and GP, and rerun GP on the Makona dataset. We note that MrBayes posterior samples on each dataset required multiple days. The repository includes copies of the posterior samples for reproduction without requiring the full MrBayes runs. Additionally, the repository includes copies of the raw output after VBPI and GP estimation used for figures and results presented in this manuscript.

## Discussion

In this paper, we extend the Felsenstein pruning algorithm to a multi-tree phylogenetic model represented with a subsplit DAG. By using a dynamic program, we calculate partial likelihood vectors on the internal nodes of the DAG by marginalizing over topologies and molecular character states. This enables efficient calculation of a composite-like marginal likelihood that represents the per-site conditional probability of states given branch length parameters on the topologies observed in the DAG. We modify this likelihood by instead only marginalizing over topologies that contain a specific edge, defining the per edge marginal likelihood and the per edge composite likelihood under a standard assumption of independence over sites. The per edge composite likelihood serves as the objective function that allows us to make point estimates for branch lengths on the DAG edges. Under a Bayesian setting, these estimates were previously only accessible through a long MCMC run or, more recently, from related work on variational Bayesian phylogenetic inference [9].

We find that the branch length point estimates we obtain from generalized pruning accord with those obtained from a MrBayes posterior sample on standard phylogenetic datasets. Greater accordance is seen when we subset on edges that appear in a greater number of posterior topologies. Generalized pruning thus trades off accuracy on the low posterior probability edges for speed, leading to estimates in seconds compared to the hours required for convergence of MCMC.

One clear application to the branch length point estimates is for parameter initialization in variational Bayesian phylogenetic inference. Under that framework, branch lengths are estimated from a log-normal distribution with mean and variance parameters that are randomly initialized and updated during stochastic gradient descent. By replacing this random initialization with the point estimates obtained from generalized pruning, we should see faster convergence of the branch length variational distributions. In practice, the mean and variance parameters for a parent–child subsplit pair are themselves parameterized by subsplit-specific components. It remains to be determined how to appropriately initialize these subsplit components such that they result in edge branch length estimates given by the generalized pruning estimates.

The generalized pruning algorithm is, to our knowledge, the first algorithm to marginalize over tree topologies in a dynamic program. However, there are aspects of this work that connect with the “structural EM” algorithm [29], which leveraged the Chow-Liu approach [30] for phylogenetics. Specifically, that work approximates the full likelihood with the product of quantities, each of which can be evaluated edge-wise. Generalized pruning also enables per-edge optimization. However, the structural EM algorithm calculates an expected log-likelihood score marginalizing over ancestral states for pairs of nodes connected by a current tree, whereas generalized pruning marginalizes over alternative topologies and ancestral states simultaneously.

Our next step is to use generalized pruning as a means of inferring the subsplit DAG itself. In the same way that maximum-likelihood algorithms consider tree arrangements following nearest-neighbor interchanges (NNIs) to find the maximum-likelihood phylogeny, we may use a similar strategy for considering NNIs on the subsplit DAG. In this case, the per edge composite likelihoods could be used to accept or reject NNIs to append onto an initial DAG built from a small number of high likelihood topologies.

We also note that there is no obstruction to using non-reversible models for this algorithm— we do not need nor use the pulley principle— and the algorithms of [17] could be adapted here as well.

## Supplementary Information

**Additional file 1: Figure S1.** Scatter plot of VBPI estimates vs MrBayes posterior means onthe branch lengths for DS1, DS3, DS4, DS5, DS6, DS7, and DS8 for sDAG edges that appear in at least 10 posterior samples.  **Figure S2.** Scatter plot of VBPI estimates vs MrBayes posterior means on the branch lengths with outlier estimates removed for DS1, DS3, DS4, DS5, DS6, DS7, and DS8.

## Data Availability

The implementation of the generalized pruning algorithm is available in our Python-interface C++ library bito (https://github.com/phylovi/bito). All data, scripts, and instructions for reproducing the results presented are available at https://github.com/matsengrp/gp-benchmark-1.
