# Supplementary material for: A topology-marginal composite likelihood via a generalized phylogenetic pruning algorithm
Source: Algorithms Mol Biol. 2023 Jul 31;18:10. doi: 10.1186/s13015-023-00235-1 (PMC10391877; doi:10.1186/s13015-023-00235-1)
Supplement: Supplementary file 1 — Additional file 1: Figure S1. Scatter plot of VBPI estimates vs MrBayes posterior means onthe branch lengths for DS1, DS3, DS4, DS5, DS6, DS7, and DS8 for sDAG edges that appear in at least 10 posterior samples. Figure S2. Scatter plot of VBPI estimates vs MrBayes posterior means on the branch lengths with outlier estimates removed for DS1, DS3, DS4, DS5, DS6, DS7, and DS8. [file 13015_2023_235_MOESM1_ESM.pdf]

Supplementary Materials

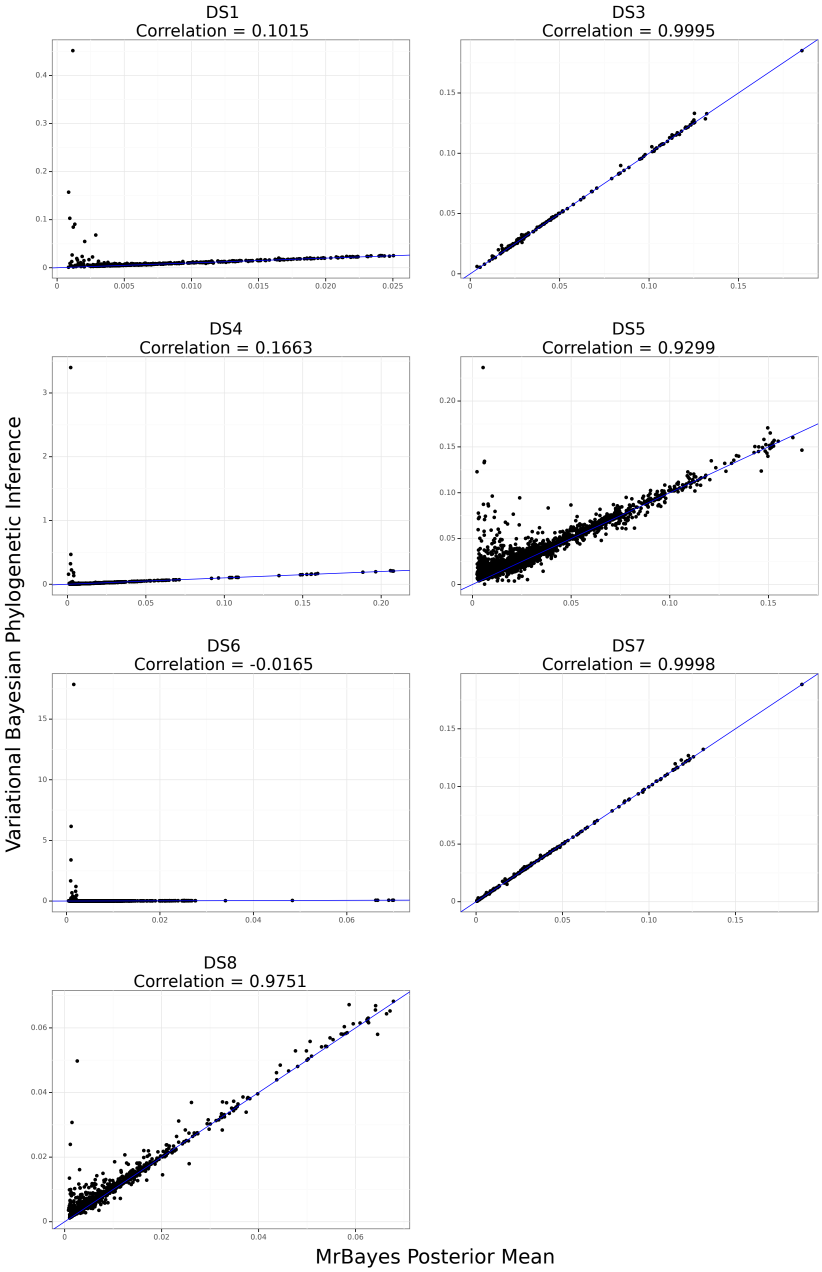

Figure S1: Scatter plot of VBPI estimates vs MrBayes posterior means on the branch lengths for DS1, DS3, DS4, DS5, DS6, DS7, and DS8 for sDAG edges that appear in at least 10 posterior samples. The MrBayes posterior was sampled with a Exponential(10) prior on branch lengths.

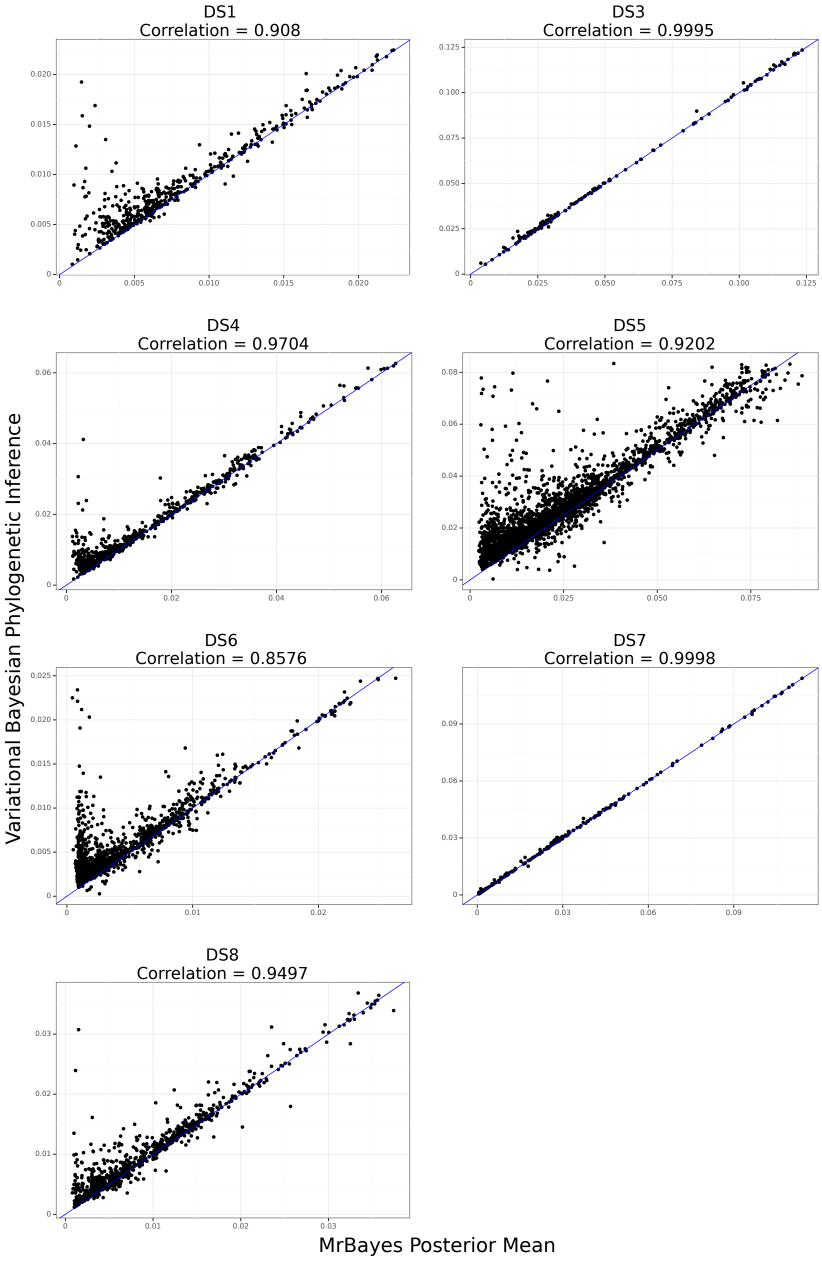

Figure S2: Scatter plot of VBPI estimates vs MrBayes posterior means on the branch lengths with outlier estimates removed for DS1, DS3, DS4, DS5, DS6, DS7, and DS8. Outliers were defined as VBPI estimates which fell greater than the 95% quantile in each dataset.
